# Carbon bursts inside a small aperture intraocular lens after Nd:YAG laser capsulotomy

**DOI:** 10.1016/j.ajoc.2024.102129

**Published:** 2024-07-21

**Authors:** Julian Bucur, Liliana Werner, Thomas Kohnen

**Affiliations:** a Department of Ophthalmology, Goethe-University, Frankfurt am Main, Germany; b John A. Moran Eye Center, Intermountain Ocular Research Center, University of Utah, Salt Lake City, USA

**Keywords:** Small aperture IOL, Pinhole IOL, EDOF, Nd:YAG laser capsulotomy, Optical phenomena

## Abstract

**Purpose:**

The IC-8® Apthera™ (AcuFocus Inc.™, Irvine, California, USA) is the first small aperture intraocular lens (IOL) to receive FDA approval for presbyopia correction in the summer of 2022. It is a single-piece hydrophobic acrylic monofocal lens, which is placed in the capsular bag. In its center it carries a black circular mask (FilterRing™) with a diameter of 3.23 mm consisting of polyvinylidene fluoride and carbon black nanoparticles. In the center of this mask sits a 1.36 mm wide aperture. Thanks to this pinhole effect the IC-8® serves as an extended-depth-of-focus (EDOF) IOL and can be used in presbyopia correction.

This report describes the case of a patient with an IC-8® implant who underwent Nd:YAG laser capsulotomy for posterior capsule opacification (PCO). The post laser checkup showed a dark central optical change within the IOL and the patient described optical phenomena as well as blurred central vision, which is why he received IOL exchange. The explanted IC-8® was sent to the Intermountain Ocular Research Center at the University of Utah for further analysis.

**Observations:**

A 56-year-old male underwent cataract surgery with implantation of a non-diffractive EDOF-IOL on the right and the IC-8® small aperture IOL on the left eye. On the left eye, the patient had received penetrating keratoplasty seven years prior to the cataract operation due to posttraumatic corneal scarring. The early checkups after cataract surgery showed a corrected distance visual acuity (CDVA) in the left eye of +0.1 logMAR in the first month. About 5 months after the operation, PCO was first described on the left eye leading to a decrease in visual acuity to +0.4 logMAR (CDVA). Due to PCO, Nd:YAG laser capsulotomy was conducted 5 months after the cataract operation on the left eye. 12 shots were applied at 2.7 mJ. The following appointments showed a continuously reduced visual acuity of +1.3 logMAR (uncorrected) on the left eye and the patient described blurry and ‘swirled’ central vision. By slightly tilting his head and thus not using the center of his optic axis, he would be able to see sharper. Slit lamp examination showed a small optical change inside the IC-8® IOL not resembling a pit but believed to be a small pocket of air. Due to the ongoing symptoms as well as the reduced VA, the seemingly damaged small aperture IOL was exchanged for a three-piece hydrophobic acrylic monofocal lens, which was also placed in the posterior chamber. The explanted IC-8® was sent to the Intermountain Ocular Research Center at the University of Utah for further analysis. Results from gross and light microscopic analysis showed that the change caused by the Nd:YAG laser application consisted of a localized optical area containing carbon black nanoparticles used for the circular mask within the IOL.

**Conclusions and importance:**

When dealing with PCO and performing Nd:YAG laser capsulotomy in eyes with an IC-8® IOL implant, the laser shots should be applied either inside the aperture or outside of the black circular mask of the IOL. Otherwise, the Nd:YAG laser can lead to bursts of carbon nanoparticles within the IOL which may cause optical phenomena as well as decreased visual acuity possibly resulting in an IOL exchange.

## 1 Case report

A 56-year-old male visited our department for planning cataract surgery in both eyes. His ophthalmic history consisted of penetrating keratoplasty on the left eye, which was done seven years prior due to posttraumatic corneal scarring. This case report is centered on the postoperative development on the left eye.

## 2 Clinical examination

The examination revealed a bilateral cataract. The corrected distance visual acuity on the right eye was +0.3 logMAR with −11.5/−1.5/104° (uncorrected: +1.3 logMAR) and on the left eye it was +0.1 logMAR with +0.75/−2.0/97° (uncorrected: +0.2 logMAR).

A Scheimpflug examination (Pentacam AXL, Oculus, Wetzlar, Germany) showed an anterior chamber depth of 2.93 mm on the right and 3.06 mm on the left eye. Total corneal refractive power (TCRP) in the 3 mm zone was 1.3 D at an axis of 168° on the right and 2.4 D at an axis of 108° on the left eye.

Biometry (IOL-Master 700, Zeiss Meditec, Jena, Germany) showed the axial length was 24.89 mm with an astigmatism of −0.34 D at an axis of 159° on the right eye and 24.80 mm with an astigmatism of −3.29 D at an axis of 153° on the left eye.

Endothelial cell count was 2226 cells/mm^2^ in the right and 752 cells/mm^2^ in the left eye.

The clinical examination, using a slit lamp, showed nuclear sclerosis of the lens with deep anterior chambers in both eyes. The left eye showed a clear corneal transplant without any signs of graft rejection or failure. Fundus examination was unremarkable for pathological findings.

## 3 Preoperative planning

The patient wished for spectacle independence in far and intermediate distances because he was still driving quite often. Due to the corneal irregularity in the patient's left eye after the previous penetrating keratoplasty, the decision was made to implant a monofocal pinhole IOL creating an EDOF effect.^1^ Thus, the IC-8® was chosen and calculated according to the biometry of the patient's left eye.

## 4 Operation

On the left eye, through a posterior limbal incision the IC-8® Apthera™ IOL (+20.0 D) (AcuFocus Inc.™, Irvine, California, USA; acquired by Bausch & Lomb in 2023) was implanted in the capsular bag manually, as the patient did not wish for laser assisted surgery. The operation was performed in local anesthesia by an experienced surgeon (T.K.).

## 5 Postoperative examinations

In the first weeks after cataract surgery, the visual acuity of the left eye remained stable at a CDVA of +0.1 logMAR with +0.75/−3.0/90° (UDVA: +0.3 logMAR) and the patient also noticed an improvement. After 5 months, the CDVA in the left eye decreased to +0.4 logMAR with +1.25/−4.5/90° (UDVA: +1.1 logMAR) and PCO was diagnosed. Hence, Nd:YAG laser capsulotomy was conducted on the left eye by the same experienced surgeon who had done the cataract surgery (T.K.). 12 shots were applied at 2.7 mJ.

Following the standard laser capsulotomy procedure for EDOF lenses, the circular PCO target area was kept narrow and mostly within the borders of the black circular FilterRing™ mask. However, one of the laser shots caused an IOL pit. The intraocular pressure was normal afterwards and a non-steroidal anti-inflammatory eye drop was prescribed for one week.

The checkup one month after the laser capsulotomy on the left eye showed an optical change enclosed within the black circular mask of the IOL, resembling an entrapped bubble of air. The UDVA at this point was +1.3 logMAR with the refraction not being measurable because the patient described blurry and ‘swirly’ central vision, which became clearer by tilting his head and looking outside of the central axis. Due to this IOL optic change sitting slightly outside the IOL optic center, the checkups were continued for the moment, and no surgical intervention was made at this point.

Four months after the laser capsulotomy, the patient returned for a checkup and still described blurred central vision in his left eye. By slightly turning his head, he would still be able to see sharper with his left eye. His CDVA remained at +1.3 logMAR and therefore, in unison with the patient, the decision was made to exchange the IC-8® (still showing the burst inside) for a monofocal IOL (+18.0 D).

## 6 Intraocular lens exchange

The exchange was performed 9 months after the initial implantation and 4 months after Nd:YAG laser posterior capsulotomy. After bisection, the IC-8® IOL (+20.0 D) was removed through a 3 mm incision and an AcrySof® (Alcon™, Fort Worth, Texas, USA) MA60AC (+18.0 D) was implanted in the capsular bag without any complications and by the same experienced surgeon as the first operation and the laser capsulotomy. Further postoperative care was carried out to the clinical standardized guidelines.

**Fig. 1:**
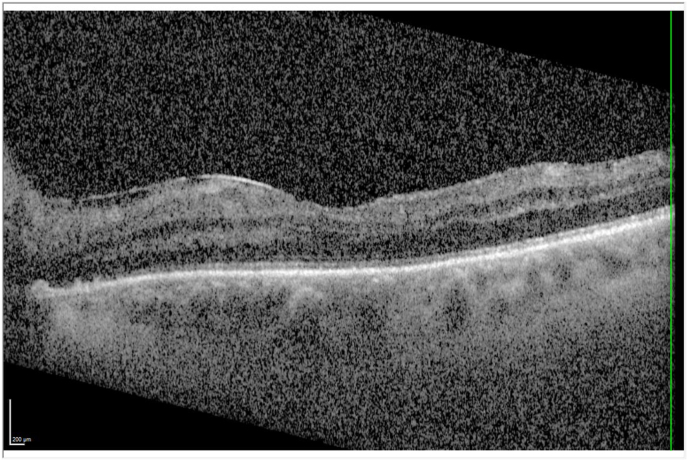
OCT of the left eye two weeks after lens exchange.

Two weeks after the exchange, the left eye showed an increased uncorrected distance visual acuity (UDVA) of +1.0 logMAR. CDVA stayed the same at +1.0 logMAR. OCT showed an epiretinal membrane with slight consecutive retinal thickening (Fig. 1). The patient reported slightly sharper central vision and no remaining optical phenomena.

One month after the exchange, the left eye showed an increased uncorrected distance visual acuity (UDVA) of +0.9 logMAR. The CDVA was also at +0.9 logMAR. OCT images were stable to the prior visit.

**Fig. 2:**
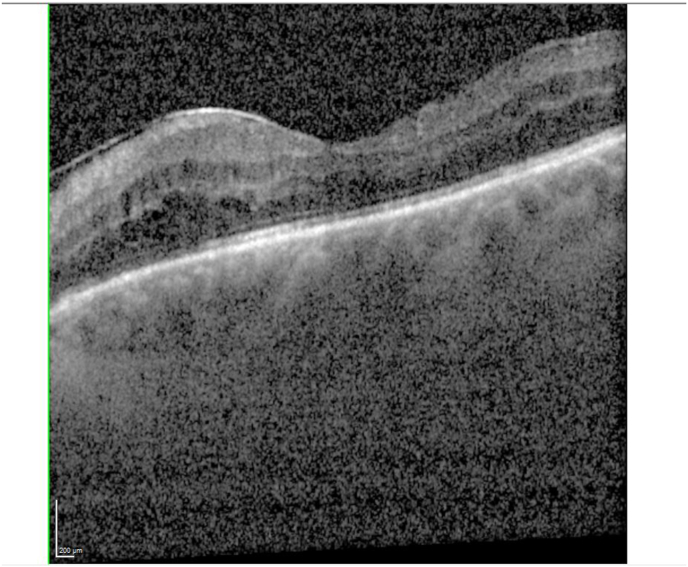
OCT of the left eye six months after lens exchange.

Six months after the exchange, the left eye showed a slight deterioration in UDVA and CDVA to +1.3 logMAR. On this visit, OCT showed macular edema alongside the aforementioned epiretinal membrane (Fig. 2). Thus, a topic therapy with dorzolamide and ketorolac was initiated.

**Fig. 3:**
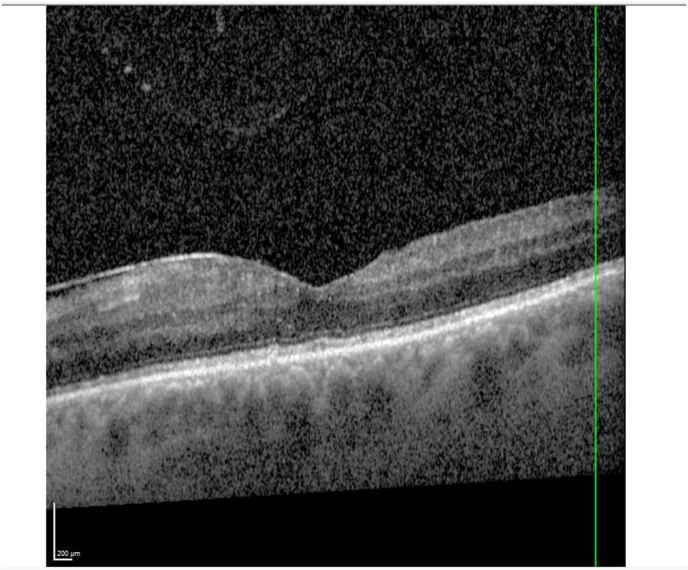
OCT of the left eye seven months after lens exchange.

Seven months after the exchange, UDVA was at +1.0 logMAR while CDVA (+1.0/−5.0/92°) had improved to +0.3 logMAR. Macular edema was no longer present in OCT images, with only singular intraretinals cysts remaining (Fig. 3).

**Fig. 4:**
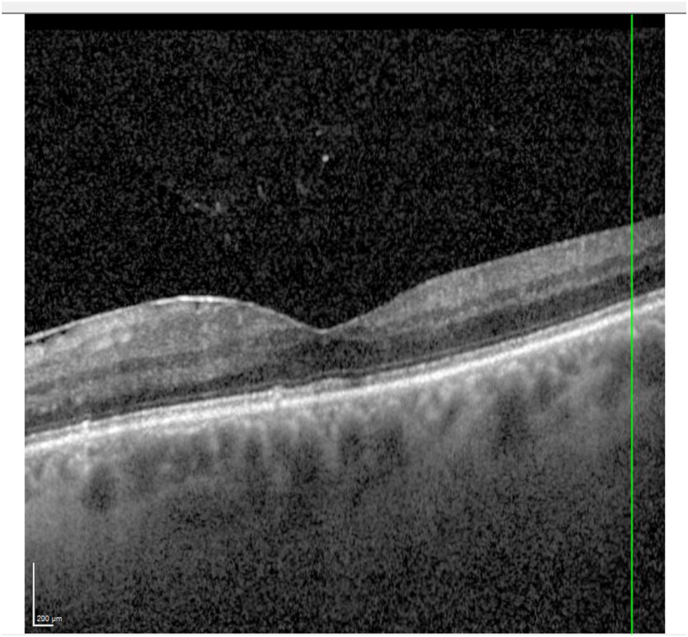
OCT of the left eye eight months after lens exchange.

Eight months after the exchange, UDVA was still at +1.0 logMAR. CDVA (+1.0/−5.0/92°) had improved further to +0.2 logMAR. OCT images showed a stable epiretinal membrane with singular intraretinals cysts remaining (Fig. 4).

## 7 Laboratorial analyses

**Fig. 5:**
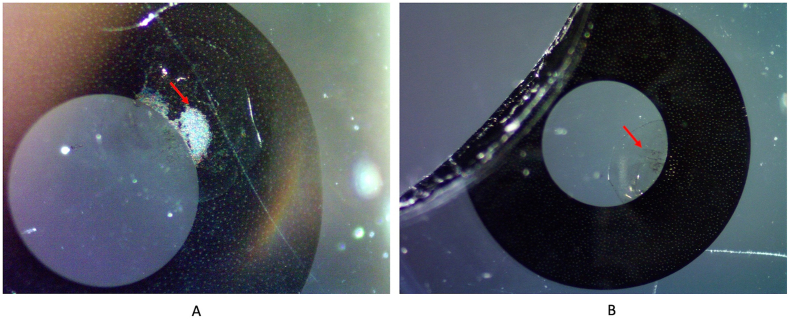
Gross photographs of the explanted Apthera IOL taken from an anterior (A) and a posterior (B) view. The arrow in A shows the localized area of discoloration of the black mask. The arrow in B shows multiple black pigments dispersed within the optic of the lens, adjacent to the mask discoloration.

**Fig. 6:**
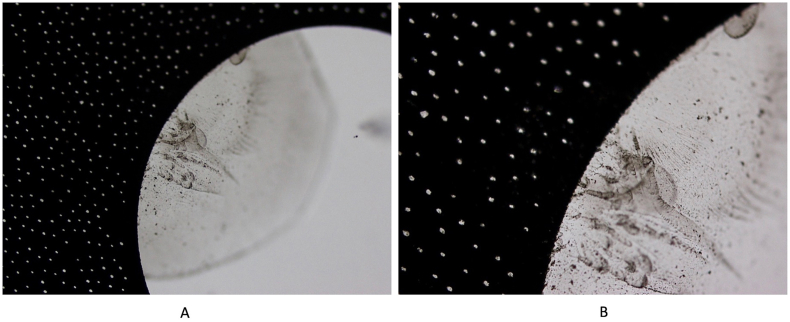
Light photomicrographs of the explanted Apthera IOL. The black pigments dispersed within the optic likely correspond to carbon black nanoparticles from the mask of the lens. A and B, original magnification X200 and X400, respectively.

Gross examination of the bisected explanted IOL was performed, and gross pictures were taken using a Nikon digital camera (model D1x with a Nikon ED 28–70 mm AF lens). Microscopic examination and photographs under a light microscope (Olympus, Optical Co. Ltd) were also completed. They showed a localized area of discoloration close to the inner edge of the intra-optical black mask. Adjacent to this area, the optic of the lens exhibited an overall circular area containing multiple black pigments dispersed within it, likely corresponding to carbon black nanoparticles from the mask of the lens (Fig. 5, Fig. 6).

## 8 Discussion

In patients with corneal irregularities (e. g. after penetrating keratoplasty or corneal scarring), who wish for spectacle independence in far and intermediate distances in particular, small aperture IOLs like the IC-8® have become viable options when treating cataract or pursuing refractive lens exchange.^2^, ^3^, ^4^

Monocular implantation of the IC-8® also showed good visual acuity results in near distances, as proven in studies going back to 2015.^5^

Nonetheless, small aperture IOLs are not exempt from possible PCO, which can lead to optical phenomena as well as an overall decrease in visual acuity. The standard treatment for PCO is capsulotomy using a Nd:YAG laser. In most cases, it is preferred over a surgical approach as it is a noninvasive, quicker and safer procedure when performed by an experienced surgeon.

However, Nd:YAG laser capsulotomy is also not exempt from possible complications. These include pits within the IOL, increased intraocular pressure (IOP), cystoid macular edema, retinal detachment, vitreous prolapse, corneal injury, full thickness IOL defects and IOL movement with consecutive refractive changes.^6^, ^7^, ^8^, ^9^ Two variables of the procedure that are regarded as the main influences on the rate of intraocular complications are the amount of used laser energy and the size of the capsulotomy. In most studies, higher levels of laser energy as well as larger capsulotomies were associated with increased IOP and macular thickness,^8^ while some studies did not identify the level of laser energy as a risk factor for complications.^6^

When performing Nd:YAG laser capsulotomy on EDOF lenses, most surgeons still prefer a smaller capsulotomy. Nevertheless, when a patient with an IC-8® implant is scheduled for PCO treatment, the laser technique and the capsulotomy pattern must be adjusted to the characteristics of the IOL.

Our case shows that when performing Nd:YAG laser capsulotomy on patients with an IC-8® lens implant, the surgeon has to keep his laser spots away from the inner circular mask of the IOL, consisting of carbon nanoparticles. In the event of setting laser shots too close to that mask, carbon bursts can appear within the IOL, which can lead to optical phenomena, deteriorated visual acuity and ultimately lens exchange.

## 9 Conclusion

**Fig. 7:**
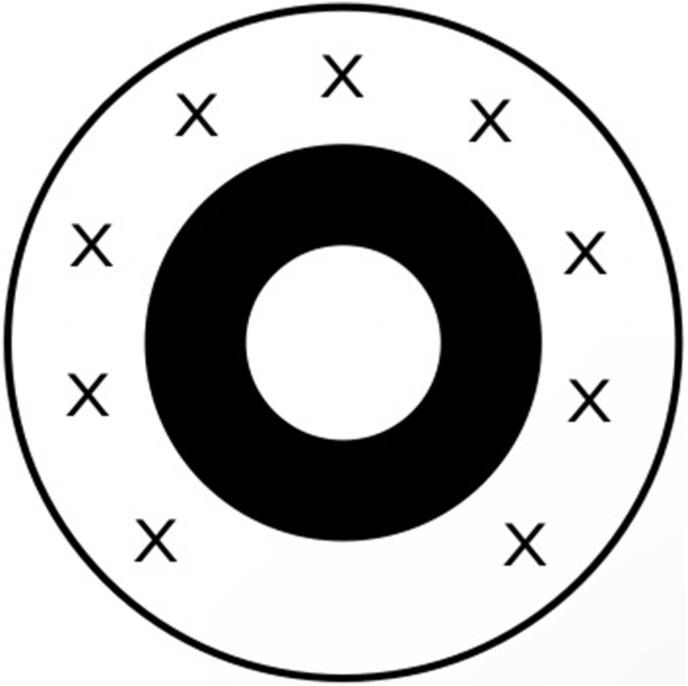
Scheme of preferred laser capsulotomy pattern in patients with an IC-8® implant. Laser shots should be applied outside of the circular carbon mask (black marks).

When performing Nd:YAG laser capsulotomy in patients with an IC-8® implant and PCO, one of two laser techniques are advised: either staying inside the central aperture or outside of the circular carbon mask incorporated within the IOL. Bausch & Lomb recommends placing laser shots approximately 1.0 mm outside of the carbon mask and following a capsulotomy pattern with an inferior hinge (Fig. 7). Higher proximity of laser shots to the circular mask may cause intra-optic carbon bursts, which may require lens exchange due to subjective visual disturbances noticed by the patient.

## Patient consent

The patient consented to publication of the case and the images in writing.

## Funding disclosures

This work was supported in part by the National Institutes of Health Core Grant (EY014800), and an Unrestricted Grant from Research to Prevent Blindness, New York, NY, to the Department of Ophthalmology and Visual Sciences, University of Utah.

## Authorship

All authors attest that they meet the current ICMJE criteria for Authorship.

## CRediT authorship contribution statement

**Julian Bucur:** Writing – review & editing, Writing – original draft, Methodology, Formal analysis, Data curation, Conceptualization. **Liliana Werner:** Writing – original draft, Methodology, Formal analysis. **Thomas Kohnen:** Writing – review & editing, Supervision, Project administration, Methodology, Conceptualization.

## Declaration of competing interest

The authors declare the following financial interests/personal relationships which may be considered as potential competing interests:Thomas Kohnen reports a relationship with Alcon Laboratories Inc that includes: consulting or advisory. Thomas Kohnen reports a relationship with Johnson & Johnson Surgical Vision Inc that includes: consulting or advisory. Thomas Kohnen reports a relationship with LensGen that includes: consulting or advisory. Thomas Kohnen reports a relationship with Oculentis that includes: consulting or advisory. Thomas Kohnen reports a relationship with Oculus that includes: consulting or advisory. Thomas Kohnen reports a relationship with Presbia that includes: consulting or advisory. Thomas Kohnen reports a relationship with Schwind that includes: consulting or advisory. Thomas Kohnen reports a relationship with Carl Zeiss Meditec Inc that includes: consulting or advisory. Thomas Kohnen reports a relationship with Allergan that includes: consulting or advisory. Thomas Kohnen reports a relationship with Bausch & Lomb that includes: consulting or advisory. Thomas Kohnen reports a relationship with Geuder that includes: consulting or advisory. Thomas Kohnen reports a relationship with MedUpdate that includes: consulting or advisory. Thomas Kohnen reports a relationship with SANTEN that includes: consulting or advisory. Thomas Kohnen reports a relationship with Staar that includes: consulting or advisory. Thomas Kohnen reports a relationship with Thieme that includes: consulting or advisory. Thomas Kohnen reports a relationship with Ziemer that includes: consulting or advisory. If there are other authors, they declare that they have no known competing financial interests or personal relationships that could have appeared to influence the work reported in this paper.
